# An Approach for Investigating Sexual Maturity in Wild Boar Males: Testosterone and 17β-Estradiol Analysis

**DOI:** 10.3390/ani12172295

**Published:** 2022-09-05

**Authors:** Claudia Maistrelli, Marion Schmicke, Martina Hoedemaker, Ursula Siebert

**Affiliations:** 1 Institute for Terrestrial and Aquatic Wildlife Research, University of Veterinary Medicine Hannover, Foundation, 30173 Hannover, Germany; 2 Clinical Endocrinology Laboratory, Clinic for Cattle, University of Veterinary Medicine Hannover, Foundation, 30173 Hannover, Germany; 3 Clinic for Cattle, University of Veterinary Medicine Hannover, Foundation, 30173 Hannover, Germany

**Keywords:** testosterone, 17β-estradiol, wild boar, reproduction, puberty

## Abstract

**Simple Summary:**

Wild boar numbers have increased constantly over the last three decades, as have their related conflicts with modern society. For this reason, wild boars have become a frequent topic of discussion. This article describes and explains the concentrations of the two main sexual steroids, testosterone and estradiol, as well as our effort to define threshold levels to recognize the sexual maturation of wild boar males and differentiate between immature and adult boars. With the help of additional analyses on spermatozoa and morphological data, it was shown that prepubertal boars at 8 months of age could be clearly differentiated from postpubertal boars, showing significantly lower concentrations of hormones. Wild boar males older than 11 months of age, in contrast, presented hormonal values like adults. Before the completion of their first year, wild boars are physiologically able to reproduce and may take part in reproduction. The high hormonal concentrations at the end of the breeding season suggest that the mating period could last longer than usually supposed, shifting farrowing into the summer.

**Abstract:**

Analyses of sexual steroid hormones in wild boars are rarely described. Testosterone (T) and 17β-estradiol (E2) concentrations are useful to recognize sexual maturation. As threshold values for this species are unknown, additional parameters are required. A total of 127 blood samples from wild boar males were collected to measure T and E2. Age and weight were recorded. Thirty-one epididymides were sampled too. Males were sorted into pre-and postpubertal groups based on the absence/presence of spermatozoa in epididymides and on morphological data following previous results. Forty-four males were prepubertal: the mean age and weight were 10 months and 23 kg, respectively. They showed no spermatozoa. The mean concentrations of T and E2 were 1.2 ± 1.2 ng/mL and 39.7 ± 120.3 pg/mL, respectively. Sixty-six males were postpubertal, twenty-nine of which presented spermatozoa. Their mean concentration of T was 7.6 ± 6.3 ng/mL and E2 was 664.3 ± 250.4 pg/mL. Seventeen samples could not be defined; the hormone concentrations between the two groups suggested a transitional phase consistent with puberty. Wild boars before 12 months of age had high hormone levels like older boars, indicating that they could attempt to reproduce. Hormones at the end of the mating season (January) were high so that reproduction could occur thereafter, shifting farrowing from spring to summer.

## 1. Introduction

The wild boar (*Sus scrofa*) population has increased steadily since the 1980s [1]. Despite high mortality rates, the population can recover fast because of the high reproductive potential of this species. Therefore, understanding reproduction dynamics is necessary to control the local population numbers successfully, which is of significant interest, considering the current spread of African swine fever in some European countries [2]. One tool for evaluating the reproductive functions in a wild population is to assess the endocrine activity through hormone measurements in either blood, feces, saliva, or other material [3]. Endocrine analyses of steroid hormones enable us to evaluate the reproductive status of animals; however, there is not much information about sex hormone concentrations for the wild boar [4,5].

In Germany, the wild boar breeding season occurs between autumn and winter, i.e., from about October until January [6] when the day length is shortened, with farrowing approximately four months later between the end of winter and spring, which coincides with February until May. Recent investigations showed that piglets of both sexes reach puberty from about six to nine months of age and sexual maturity already in their first year of life; however, almost none of these studies evaluated sex hormones [6,7,8]. In fact, puberty was assessed macro- and microscopically in wild boar females by determining the diameter and presence of follicles/corpora lutea, i.e., the stage of the estrus cycle [6,9], whereas in males, it was determined by assessing the stage of spermatogenesis by means of histology and immunohistochemistry of the anti-Müllerian hormone and androgen receptor [8]. In the rut season, wild boar males of the previous year (sub-adults, i.e., from 12 to 24 months of age) and adults (over 2 years of age) attempt to reproduce. Juveniles (under 12 months of age), which passed puberty during the breeding season develop sexual maturation at about the end of the breeding season, consequently [8].

Sexual maturation, and, therefore, the completion of spermatogenesis with production of spermatozoa, is driven by androgens [10]. The production of androgen, of which testosterone is the main male steroid hormone, occurs in Leydig cells and is stimulated by the luteinizing hormone [10]. Testosterone acts on the androgen receptor localized in the Sertoli cells and promotes the development of sex organs, normal reproductive function, and sexual behavior, among others [11]. Testosterone, therefore, can be used to monitor the reproductive state as well as the achievement of puberty, as its levels are low before puberty and rise thereafter [10]. However, to the best of the authors’ knowledge, assessing testosterone concentrations in wild boars in order to determine the achievement of sexual maturation has only been conducted in one French study in the 1980s [5].

The hormone 17β-estradiol derives primarily from the aromatization by D-aspartic acid of testosterone in the testicular tissue [12,13] and it is the main active estrogen in males that can be measured in peripheral blood [14]. The estrogen synthesis in boars is peculiar [15], as it is known to be remarkably higher than in other mammalian males. Estrogens are required for many biological functions in a synergic way with testosterone for spermatogenesis, modulation of libido, stimulation of accessory sex glands, and erectile function [14,16,17,18]. In domestic boars, estrogen binding receptors (ERα and ERβ) were reported in the testis on Sertoli cells, Leydig cells, and germ cells until spermatids, with different percentages of expression of both receptors depending on the phase of sexual development [19,20]. It is reported that absence or dysfunction of the ERα resulted in disruption of sperm morphology, affected negatively fertility, etc. [21]. An impairment of sexual behavior with reduction in mounting frequency was observed in animal models with estrogen deficiency [22]. However, the exact role of estrogen receptors in the testis is not fully understood yet [23] and localization, as well as the presence of both or only one of the estrogen receptors, differ between mammalian species [21]. Moreover, the complex mechanism related to the function of D-aspartate in regulating steroidogenesis in Leydig cells, such as the activation or deactivation of the androgenic or the estrogenic pathway during breeding and non-reproductive periods as well as in a-seasonal species such as the domestic pig, is still the focus of recent studies [13,24]. Studies on estrogens in wild boars have referred only to female estrogens [4], and, to the best of the authors’ knowledge, investigations of estradiol in male wild boars are not yet described.

With the aim to contribute to develop appropriate management strategies for controlling wild boar populations, this study wanted to investigate the reproductive functions of wild boar males of every age class, with particular focus on males of juvenile age, i.e., below one year old. Moreover, considering that little is known about when young wild boar males can attempt to reproduce, endocrine investigations of testosterone (T) and 17β-estradiol (E2) would help to estimate at which extent these males could take part in reproduction during the mating season. In particular, taking into consideration the age, weight, and stage of spermatogenesis of the animals, this study attempted to determine threshold values for both steroid hormones of pre- and postpubertal animals.

## 2. Materials and Methods

This study was performed in East Lower Saxony (Germany) in an area of approximately 4900 km^2^ in the Forestry Commissions Oerrel, Unterlüß, Wolfenbüttel, and Graf von der Schulenburg. All areas of this study are characterized by sub-continental climatic conditions and similar habitats. We received permission for sampling from the respective heads of the forestry commissions.

Three types of samples were obtained from wild boars culled during the hunting season between October and January over a two-year period from 2017 to 2019. We collected peripheral blood, sampled epididymides, and performed age and weight classification as described below.

Blood samples of 127 male individuals were collected after gutting the carcass from a large thoracic vessel or directly from the heart using EDTA 7.5 mL tubes (Kabe Labortechnik GmbH, Nümbrecht-Elsenroth, Germany). They were refrigerated until arrival at the facility (usually about 3 h). Then, they were centrifuged (12 min, 4500 rpm); 1 mL plasma was transferred into 1.5 mL tubes (Eppendorf AG, Hamburg, Germany) and stored at −32 °C until assaying. Testosterone and 17β-estradiol concentrations were determined by radioimmunoassay (RIA Testosterone direct and Ultra-sensitive estradiol RIA, Beckman Coulter, Inc., Krefeld, Germany, respectively). Laboratory analyses were conducted following instruction from the manufacturer. For validation, the same sample was linearly diluted 10×. The intra-assay coefficients of variation for testosterone and 17β-estradiol were 7.56% and 5.78%, respectively, detected by measuring one wild boar sample 20 times within one test routine. 

Furthermore, it was possible to sample epididymides from 31 out of 127 males. Obtained by flushing epididymides, we investigated the sperm fluid for reproductive parameters, e.g., presence/absence of spermatozoa, sperm motility, and sperm morphology (unpublished data). Finally, ages of the wild boars were determined by teeth examination of the lower jaw in accordance with Briedermann [25] and Heck and Raschke [26]. The wild boars were then weighed after removal of the internal organs (dressed weight). 

Samples were sorted in prepubertal and postpubertal groups, cross-checking the available collected data. According to the absence or presence of spermatozoa in the flushed sperm, these samples were considered prepubertal or postpubertal, respectively. Then, samples were sorted according to detectable testosterone and estradiol concentrations as follows: Samples whose concentrations were 0 or below the minimum detectable limit according to serial dilution, i.e., 0.3 ng/mL for T and 15.0 pg/mL for E2, were classified as belonging to prepubertal wild boars.

In addition, we also relied on the classification and results obtained in a previous publication in which the attainment of puberty was defined according to the stage of spermatogenesis [8]. We categorized males as prepubertal when the weight and the age of the animal were less than 26 kg and 8 months, i.e., 29.6 ± 3.6 kg, and 9.3 ± 1.2 months, which correspond to the limits for pubertal animals [7,8]. On the other hand, individuals whose weight and age were more than 34 kg and 10 months were defined as postpubertal, these values being typical of sexually mature individuals [5,7,8]. Thus, to sum up, the definition for prepubertal boars was the absence of spermatozoa, undetectable hormone concentrations, weight < 26 kg, and age < 8 months. Postpubertal boars were defined by the presence of spermatozoa, weight > 34 kg, and age > 10 months. We applied all criteria in samples in which data were fully available. Otherwise, samples in which this was not possible were sorted by combining the remaining data.

Statistical analyses were performed with the SAS Enterprise Guide 7.15 (SAS Institute Inc., Cary, NC, USA). One-way ANOVA was used to test differences between sampling months for both hormones and both groups. Tukey’s HSD (honestly significant difference) procedure was used for pairwise comparisons within ANOVA data. To ensure correct classification in pre- and postpubertal groups and to determine threshold hormone values, an ROC (Receiver Operating Characteristic) analysis was performed. A Student’s t-test was used for comparison between sexual maturity classes. An alpha level of 0.05 was required for statistical significance. Plots were modelled with Excel 2016 (© 2019 Microsoft Corporation, Redmond, WA, USA).

Raw data can be found in supplementary material in Table S1.

## 3. Results

According to our definition, 44 samples belonged to prepubertal and 66 to postpubertal wild boar males, respectively. Seventeen samples could not be clearly assessed. Briefly, two of the thirty-one sperm samples showed no spermatozoa in the ejaculates and were classified as prepubertal. In the remaining 29, the presence of (motile) spermatozoa was observed, and the samples belonged, consequently, to postpubertal males. In five boars, testosterone concentration was not detected at all, the same for estradiol concentrations in 28 individuals. These animals were also defined as prepubertal. Thereafter, the conditions explained above were applied to weight and age values, and all the information was put together. So, the mean dressed weight of the prepubertal wild boars was 23.3 kg. Forty (91%) of the forty-four samples belonged to boars aged less than 12 months (juveniles), fitting consequently in the prepubertal classification, but the remaining four belonged to sub-adults (between one and two years of age). However, two of these sub-adults presented weights much lower than the average (20 and 10 kg, respectively) and the other two had undetectable hormone values. Therefore, forty-four samples were of prepubertal wild boars and hormone levels could be evaluated. Their mean T value was 1.2 ng/mL and the mean E2 value was 39.7 pg/mL (Table 1). Interestingly, seven of the males defined as prepubertal showed T concentrations greater than 2.0 ng/mL (mean = 3.7 ng/mL). Nevertheless, in three of them, E2 concentration was undetectable (below 15 pg/mL). In another three, the values were just measurable between 15.5 and 86.0 pg/mL, and of these, the one with the highest value (86.0 pg/mL) did not show spermatozoa at the ejaculate analysis. A relatively high E2 concentration (on average 190.0 pg/mL) was detected in seven prepubertal samples, but the weight of the individuals was just 24 kg. Finally, one prepubertal individual had an E2 value greater than 790.0 pg/mL but showed neither detectable testosterone concentrations nor spermatozoa in the ejaculate. We did not observe any significant differences between sampling months and years for this group, neither in T nor in E2 concentrations.

**Figure 1:**
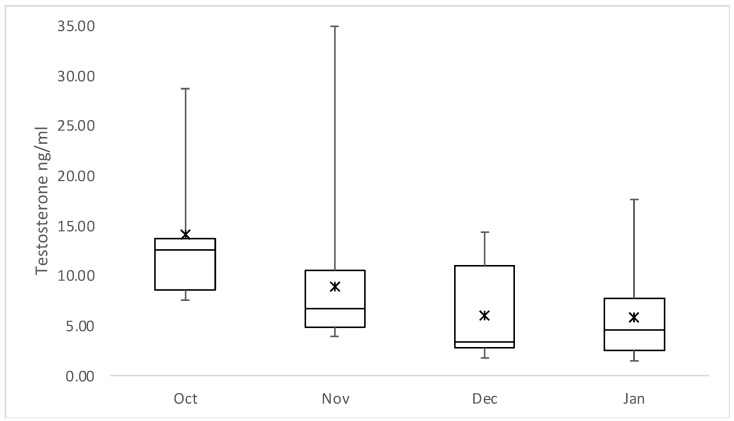
Comparison of testosterone concentrations in ng/mL between months in postpubertal wild boar males. At the beginning of the mating season in October (Oct), T values were significantly higher than in December (Dec) and/or January (Jan). Nov = November.

Sixty-six samples belonged to postpubertal wild boar males according to our definition (i.e., presence of spermatozoa in sperm fluid, >34 kg bodyweight, >10 months of age). On average, the concentration of T was 7.6 ng/mL and E2 was 664.0 pg/mL. More specifically, in 46 (70%) of the 66 samples, E2 levels were at or above the detectable upper limit of the RIA (790 pg/mL). The average weight and age of postpubertal males was 60.2 kg and 20 months, respectively, although biometric data were not available for all samples (for weight, only 56 out of 66, and age for 39 of them), or age was estimated only approximately in the absence of complete teeth eruption data. Interestingly, the age classification of these animals revealed that 14 were adults, 28 were sub-adults, and 10 were juveniles; the latter, however, were approximately 11 months old and had an average weight of 37.1 kg, fitting, therefore, in the postpubertal category. Nevertheless, we observed no significant differences in T or E2 values between the postpubertal animals of different ages, as the concentration of T was on average 7.1 ng/mL for adults, 8.6 ng/mL for sub-adults, and 5.6 ng/mL for juveniles, and for estradiol, 712.9 pg/mL, 687.1 pg/mL, and 479.7 pg/mL, respectively. In contrast, one-way ANOVA showed a significant difference (*p*-value = 0.026) in testosterone levels between the sampling months (Figure 1). These significant differences were reported by Tukey’s HSD procedure between the months of October (mean = 14.0 ng/mL) and December (mean = 6.0 ng/mL) (*p* = 0.014), as well as between the months of October (mean = 14.0 ng/mL) and January (mean = 5.8 ng/mL) (*p* = 0.01).

**Figure 2:**
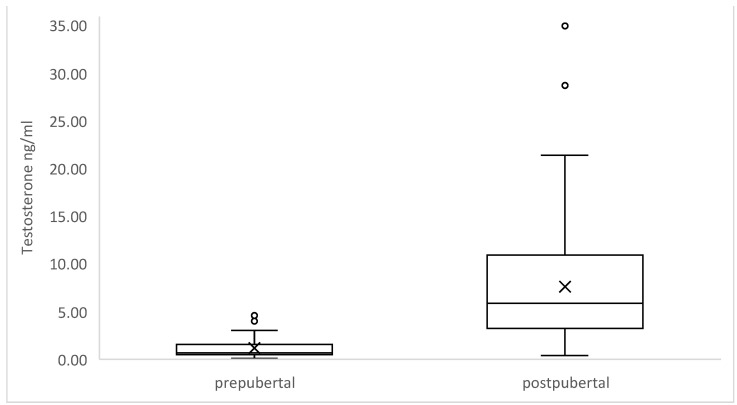
Prepubertal wild boars showed significantly lower T concentrations than postpubertal. The latter presented a wider range of values in comparison to prepubertal ones.

**Figure 3:**
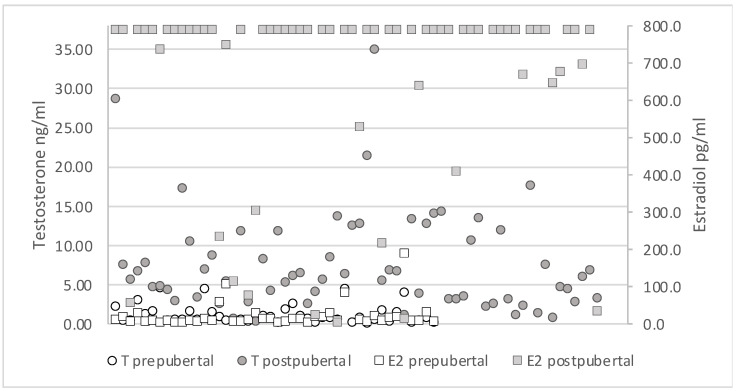
Overview of T and E2 levels of pre- and postpubertal wild boar males based on the definition chosen in this study. T concentrations are marked with dots and E2 with squares, respectively. Almost all prepubertal T and E2 values are concentrated on the lower limit, whereas T values of postpubertal males are distributed at higher concentrations. E2 in postpubertal males reached the upper detectable limit of the radioimmunoassay.

After splitting the data into pre- and postpubertal groups, we tested whether hormone concentrations significantly differed between the two groups. T concentrations in the 44 prepubertal boars demonstrated significantly lower values compared to those of the 66 postpubertal (*p*-value < 0.00001) (Figure 2), as was the same for E2 of the prepubertal group compared to the postpubertal ones (*p*-value < 0.00001) (Figure 3).

Furthermore, it was necessary to test if the group model, i.e., our binary classification into pre- and postpubertal groups, was appropriate, and if threshold levels could be defined. This was evaluated by performing a ROC (Receiver Operating Characteristic) analysis by assuming multiple cut-off points for T-concentrations (or threshold points) and determining which threshold was able to separate the groups. After assessing the sensitivity and specificity of the classifiers, the area under the ROC curve (AUC) was calculated. The AUC turned out to be 0.923553719 (accuracy of 95%), indicating that the model for classifying data into prepubertal and postpubertal categories was appropriate. Assuming a threshold level for testosterone of 2.5 ng/mL (mean_T_ + SD_T_), there was an 86% probability to correctly identify prepubertal boars. Values above 4.7 ng/mL were definitively corresponding to postpubertal wild boars.

Otherwise, we were not able to determine if the remaining 17 samples belonged to prepubertal or postpubertal wild boars. Based on age classification, all 17 belonged to juveniles, but the weight data were not clearly indicative, as the weight of the individuals was around 30 kg, which is approximately the weight at which animals are in pubertal development, and sperm samples were unfortunately not available. Indeed, their concentrations of T (mean = 2.1 ± 1.4 ng/mL) and E2 (mean = 83.0 ± 66.3 pg/mL) showed more or less wide ranges and were also not close to the lower or to the upper detectable limit.

## 4. Discussion

Our results showed that juvenile wild boar males of approximately 8 months of age had significantly lower testosterone and 17β-estradiol concentrations than the older animals. Wild boars > 11 months of age had higher steroid hormone levels, regardless of whether they were still in the juvenile class or older than two years of age. Particularly noteworthy, postpubertal young males denoted sufficient weight (on average 37 kg) and age (about 11 months) to have passed puberty [7,8], and this was also evidenced by the presence of spermatozoa in the epididymis, which indicates sexual maturation. Intermediate values of T and E2 were denoted by wide ranges, but any high peaks were found in the 17 animals whose age and weight could suggest the transition to puberty. Indeed, testosterone is the essential hormone to initiate puberty and its blood values depend on testicular development, puberty, sexual maturation, and normal sperm production. Here, it is evident that the evaluation of steroid hormone concentrations alone has its limits when animals are in the pubertal phase. Therefore, hormone investigations should be at least supplemented with additional parameters like ejaculate analysis or microscopic evaluation of spermatogenesis see in [8]. 

Moreover, postpubertal boars showed higher T-concentrations and generally a greater range in absolute values at the beginning of the mating season in October than in January, while E2 concentrations were almost all at the maximum levels analyzable by our assay. In seasonal breeders like the wild boar, testosterone is subject to cyclic changes, and before and during the mating season, the highest levels are reported [4,5]. This pattern of constant high levels of T during the breeding season, a wide range in adult males, and lower T levels or a reduction in the expression of the estrogen receptor (alpha) in the non-breeding season has also been reported for other seasonal breeding species [27,28]. In addition to seasonal fluctuations, daily fluctuations in testosterone secretion are also mentioned in the literature, although these are more pronounced in animals before puberty or are individual-related [29,30,31]. A peak in hormone concentrations is also reported during puberty [18,32,33], being the stimulus for Sertoli cells to complete spermatogenesis.

In this study, it was not possible to take regular blood samples of individuals to overcome the problem of detecting possible fluctuations in hormone levels, as this would have required at least the confinement of a wild animal with the risk of stress-related bias in hormonal values. Furthermore, a comparison of hormone concentrations with other studies is often difficult due to different specimens (serum, plasma, or feces) and methods used, e.g., in the choice of which RIA. Moreover, the possibility of comparing our results with those obtained from domestic pigs is reduced, as they often refer to the postnatal period until puberty, and until castration or slaughter at the latest (about 250 days or 8 months of age). Additionally, the management of pigs is standardized, in contrast to free-ranging wild boars. Nevertheless, although hazardous, results of this study are quite similar to other references that are summarized in Table 2.

With this premise, it is clear why it has not been determined “a priori”, whether low hormone levels meant that the animal was sexually immature and high levels indicated adult ones, or if this was due to normal fluctuations in hormonal concentrations, and moreover, why this study did not set “low” and “high” hormone ranges based on the literature. However, it could be based on supporting data showing that the highest concentrations of hormones were found in those individuals in which the completion of spermatogenesis and the presence of spermatozoa were observed. A similar pattern was also observed in the only two other similar studies on this species. In the study by Macchi, Cucuzza, Badino, Odore, Re, Bevilacqua and Malfatti [4], although most of the sampled animals were adults, the lowest T values were found in the animals under one year of age and only in a few adults that were under 30 months of age. Mauget and Boissin [5] found that T values remained low until puberty and rose significantly after 10 months. Similarly, Weiler, et al. [42] and also Schopper, et al. [43] observed a peak in testosterone secretion around 30–35 weeks, which corresponds to about 7–8 months of age and the time of the breeding period (around November).

Although the conclusions are similar, in the present study, no rise in hormone values was found in wild boars weighing about 30 kg and around 9–10 months of age, the period in which the transition to sexual maturity takes place, but above this age, the values were significantly higher. It was not possible to clearly define whether these animals were going through or had passed puberty. Although their hormone values were higher than those in non-mature animals, these reflected a wide range, supporting that in this period, there can be fluctuations due to various factors. Furthermore, even if in few samples, higher T-concentrations were observed in October, and the same pattern is reported also in the domestic pig both for testosterone and for estrogens [44]. Notably, concentrations in January, although lower than in the main mating period, were still high, suggesting that mating could occur after this period. In fact, some studies in wild boar reported a second peak of births during the summer months besides the main reported farrowing period in spring [45,46]. The results of this study could suggest that wild boar males are physiologically capable of taking part in reproduction after the main breeding season and therefore contributing to the observed second peak or shift of farrowing from spring to summer.

In the present study, E2 concentrations did not change significantly during the mating season or between different ages within the two classes. Similar to studies in the domestic pig, E2 levels rise with increasing age until puberty, but are not subjected to cyclic fluctuations as T is, and after sexual maturation, levels are constantly high [16,18,29,32,33]. One study on spotted seals affirmed that E2 concentrations were not suitable to assess sexual maturity, as no significant seasonal variations could be observed in relation to the age of the animals [27]. In fact, the role of estrogens in male reproduction is not fully understood yet [17], and in boars, estrogen synthesis is way higher than in other mammalian species [14,34], making comparisons with studies on mice or men difficult. Nevertheless, wild boar males showing viable spermatozoa had the highest E2 values, which is in line with the reported role of estrogens [15,17,47]. 

## 5. Conclusions

In conclusion, it could be shown that with the combination of T and E2 analysis, it is possible to ascertain the sexual development of wild boar males. However, in the pubertal phase, additional investigations such as the evaluation of spermatogenesis by histology may be required. Moreover, before completing the first year of age, wild boar juveniles as well as sub-adults already had high values of T and of E2 similar to older boars, indicating that they could potentially take part together with adult males in reproduction. Finally, steroid concentrations at the end of the mating season (January) were still high so that mating could occur thereafter, leading to a shift of the farrowing period from spring to the summer months. However, due to the lack of more information on endocrine investigations in the wild boar, it is suggested that more studies are necessary, especially examining steroid hormones or investigating the balance between T and E2 during the entire year. In addition, it would be highly interesting to investigate D-asp and aromatase activity during and after the main breeding season of wild boar, similar to studies in the domestic pig.

## Figures and Tables

**Table 1:** Mean concentrations of testosterone and estradiol, as well as weight and age data, with relative standard deviation (SD) for prepubertal and postpubertal wild boar males.

	Testosterone (ng/mL)	Estradiol (pg/mL)	Weight (kg)	Age (Months)
**Prepubertal (*n* = 44)**				
Mean	1.24	39.68 (22.2 *)	23.37 (22.62 *)	10.23 (9.42 *)
SD	1.21	120.31 (33.20 *)	6.09 (5.18 *)	3.27 (1.51 *)
Min	0.35	15.50	10.00	8.00
Max	4.63	790.00	42.00	20.00
**Postpubertal (*n* = 66)**				
Mean	7.61	664.30	60.23	20.72
SD	6.34	250.39	19.06	7.58
Min	0.38	15.40	16.00	10.00
Max	34.97	790.00	101.00	34.00

* mean and SD calculated without extreme values (outliers).

**Table 2:** Mean concentrations of T and E2 available in the open access literature for the domestic pig and the wild boar. Where present, information about the age and the respective number of sampled boars are provided as described in the studies.

Species	Testosterone (ng/mL)	Estradiol (pg/mL)	Age (months)	Number of Boars	Reference
Domestic boar	2.24	114.2	2	48	[29]
	9.95		4–5		
	15.75		6–7		
	8.66		8		
Domestic boar	4.18 ^1^	64.28 ^1^	8–12	11	[18]
	2.53 ^1^	20.42–29.68 ^1^	Peripubertal	10	
Domestic boar	5.5	80	4–5	11	[34]
Domestic boar		180	Adult		[15]
Domestic boar	8.8		3–4	12	[30]
	4.5		5		
Domestic boar		150–200	10	8	[35]
Domestic boar	2.2	64.7	8–9	16	[36]
	2.0	36.6	18–19		
Domestic boar	2.84	29.1	6		[32]
	4.7–7.3	128.7–116.0	Mature		
Domestic boar	5	50	8	6	[33]
Domestic boar	0.48			1	[37]
Domestic boar	4.0–17.4		6–9	9	[38]
Domestic boar	1.7 ± 0.3		5	18	[39]
	1.3 ± 0.3		6.5		
	1.4 ± 0.3		8		
Domestic boar	2.07–17.78		7	31	[40]
Domestic boar	2.14 ± 0.45 ^1^			8	[41]
Wild boar	2.39 ^2^		Mature	200	[5]
	0.74 ^3^				
Wild boar	3.57		7–12	97	[4]
	6.76		13–18		
	9.76		19–30		

^1^ Values converted from nmol/L to ng/mL for standard T and pmol/L to pg/mL for standard estradiol. ^2^ Values refer to the period November-March. ^3^ Values refer to the period April–October.

## Data Availability

The data presented in this study are available in supplementary material “Table S1”.

## Supplementary Materials

The following are available online at https://www.mdpi.com/article/10.3390/ani12172295/s1. Raw data are presented in supplementary material: Table S1: Raw data.
